# *Alternaria Brassicae* Induces Systemic Jasmonate Responses in Arabidopsis Which Travel to Neighboring Plants via a *Piriformsopora Indica* Hyphal Network and Activate Abscisic Acid Responses

**DOI:** 10.3389/fpls.2018.00626

**Published:** 2018-05-08

**Authors:** Khabat Vahabi, Michael Reichelt, Sandra S. Scholz, Alexandra C. U. Furch, Mitsuhiro Matsuo, Joy M. Johnson, Irena Sherameti, Jonathan Gershenzon, Ralf Oelmüller

**Affiliations:** ^1^Department of Plant Physiology, Matthias Schleiden Institute of Genetics, Bioinformatics and Molecular Botany, Friedrich-Schiller-University Jena, Jena, Germany; ^2^Department of Biochemistry, Max-Planck Institute for Chemical Ecology, Jena, Germany

**Keywords:** systemic signaling, interplant communication, REDOX RESPONSIVE TRANSCRIPTION FACTOR1, NITRATE TRANSPORTER2.5, *Piriformospora indica*, *Alternaria brassicae*, jasmonic acid, abscisic acid

## Abstract

Stress information received by a particular local plant tissue is transferred to other tissues and neighboring plants, but how the information travels is not well understood. Application of *Alternaria Brassicae* spores to Arabidopsis leaves or roots stimulates local accumulation of jasmonic acid (JA), the expression of JA-responsive genes, as well as of *NITRATE TRANSPORTER* (*NRT*)*2.5* and *REDOX RESPONSIVE TRANSCRIPTION FACTOR1* (*RRTF1*). Infection information is systemically spread over the entire seedling and propagates radially from infected to non-infected leaves, axially from leaves to roots, and *vice versa*. The local and systemic *NRT2.5* responses are reduced in the *jar1* mutant, and the *RRTF1* response in the *rbohD* mutant. Information about *A. brassicae* infection travels slowly to uninfected neighboring plants via a *Piriformospora Indica* hyphal network, where *NRT2.5* and *RRTF1* are up-regulated. The systemic *A. brassicae*-induced JA response in infected plants is converted to an abscisic acid (ABA) response in the neighboring plant where ABA and ABA-responsive genes are induced. We propose that the local threat information induced by *A. brassicae* infection is spread over the entire plant and transferred to neighboring plants via a *P. indica* hyphal network. The JA-specific response is converted to a general ABA-mediated stress response in the neighboring plant.

## Introduction

Long distance signaling and organ-to-organ communication are essential features of all plants (Huber and Bauerle, 2016). These processes allow information perceived locally to be systemically spread over the entire plant body, and integrated by regulatory networks causing non-cell autonomous responses in neighboring and systemic cells (Suzuki and Mittler, 2012; Fu and Dong, 2013; Kliebenstein, 2014). For example, after pathogen attack systemic responses can provide a memory of initial infection by priming remote leaves for enhanced defense and immunity to re-infection (Reimer-Michalski and Conrath, 2016). After colonization with beneficial root-colonizing microbes, root-to-shoot signaling and induced systemic resistance (Vlot et al., 2008) play important roles in resistance responses of the aerial parts of plants (cf. Erb et al., 2009; Pieterse et al., 2014). Numerous mobile signals have been described in these contexts. Fast information transfer is proposed to be associated with Ca^2+^, reactive oxygen species (ROS) and electropotential waves, and/or altered hydraulic pressure (Christmann et al., 2007; Miller et al., 2009; Zimmermann et al., 2009; Kudla et al., 2010; Swanson et al., 2011; Choi et al., 2012, 2016; Mousavi et al., 2013; Gilroy et al., 2014; Jayaraman et al., 2014; Steinhorst and Kudla, 2014; van Bel et al., 2014; Kiep et al., 2015; Hedrich et al., 2016). RNAs and miRNA propagate more slowly through the vascular tissue (Hannapel et al., 2013; Chien et al., 2017), while hormones, proteins, peptides, and small molecules also transfer specific information to distal areas on a slower scale (Dempsey and Klessig, 2012; Jimenez-Aleman et al., 2015; Lacombe and Achard, 2016). In Arabidopsis, systemic leaf-to-leaf signaling depends on direct vascular connections of local and systemic leaves, hard-wired by the developmental pattern of the rosettes (Dengler, 2006; Mousavi et al., 2013; Salvador-Recatalà et al., 2014; Kiep et al., 2015).

Likewise, N and P sensing activates long-distance signaling to coordinate nutrient homeostasis (Li et al., 2014; Ma et al., 2015; Okamoto et al., 2016; Puga et al., 2017; Xuan et al., 2017). Information on the status of Fe and other important ions is systemically spread over the entire plant body (Gayomba et al., 2015). Concentration gradients inform distal tissues about metabolic changes and activate transport or signaling events along these gradients. Examples are source/sink relationships for the sugar transport through the phloem (cf. Lemoine et al., 2013), or cellular nitrate gradients that activate translocation of nitrate to nitrate-deprived tissues (cf. White et al., 2016).

Besides systemic signaling within the plant body, threat information is also translocated to neighboring plants. Volatile organic compounds (VOCs) and green leaf volatiles emitted by stress-exposed plants activate the defense machinery in neighboring plants (Arimura et al., 2000; Baldwin et al., 2002, 2006; Matsui, 2006; Niinemets et al., 2013), and the VOC blend determines the specificity of interplant communications (Ueda et al., 2012). Interplant communication is also mediated by common mycorrhizal networks (CMN), which interconnects roots of the same or different plant species (Fitter et al., 1998; Giovannetti et al., 2006; Selosse et al., 2006; Simard et al., 2012). CMNs transfer threat information, but also C, N, and P from one plant to another (Leake et al., 2004; He et al., 2009; Ren et al., 2013), thereby promoting plant performance, resource distribution within communities (Eason et al., 1991; Selosse et al., 2006; He et al., 2009; Barto et al., 2012) and survival of seedlings on the forest floor (Dickie et al., 2005a,b; McGuire, 2007; Teste et al., 2009; Booth and Hoeksema, 2010; Bingham and Simard, 2011, 2012). A good candidate for interplant communication is the root-colonizing endophyte *Piriformospora indica* because it colonizes the roots of many plant species. *P. indica* promotes plant performance by supplying nutrients and conferring resistance against biotic and abiotic stresses, similar to mycorrhizal fungi of CNN (Camehl et al., 2011; Hilbert et al., 2012; Dong et al., 2013; Harrach et al., 2013; Venus and Oelmüller, 2013; Yogawat et al., 2013; Sun et al., 2014; Ye et al., 2014; Matsuo et al., 2015).

Here, we demonstrate that Arabidopsis plants infected with *A. brassicae* spores distribute the information within the entire plant body and inform neighboring non-infected plants about the threat via a *P. indica* hyphal network. The information flow was monitored by measuring *NITRATE TRANSPORTER2.5* (*NRT2.5)* and *REDOX-RESPONSIVE TRANSCRIPTION FACOR1* (*RRTF1)* mRNA levels, since these mRNAs responded systemically to *A. brassicae* infection in distal leaves and roots. NRT2.5 plays an important role in nitrate acquisition and remobilization in N-starved plants, takes part in nitrate loading into the phloem (Lezhneva et al., 2014) and together with NRT2.6 is involved in Arabidopsis growth promotion by the rhizobacterium *Phyllobacterium brassicacearum* STM196 (Mantelin et al., 2006; Dechorgnat et al., 2012; Kechid et al., 2013). The transporter is part of a complex with NRT2.1 which contributes to the high-affinity nitrate transport (Kotur and Glass, 2014). Nitrate transporters also function in nitrate sensing to coordinate distribution of this nutrient within the plant body (Chopin et al., 2007; Wang et al., 2012; Fagard et al., 2014; Krapp et al., 2014). The highly conserved RRTF1 induces ROS accumulation in response to abiotic and biotic stress signals, and the *RRTF1* mRNA is rapidly upregulated by H_2_O_2_ and other ROS, as well as biotic- and abiotic-induced redox signals (Khandelwal et al., 2008; Matsuo and Oelmüller, 2015; Matsuo et al., 2015). This transcription factor also stimulates systemic ROS accumulation in distal non-treated leaves (Matsuo et al., 2015). Our data suggest that *NRT2.5* and *RRTF1* are good marker genes to monitor local and systemic stress responses as well as the reaction in the non-treated neighboring plants. Besides, *A. brassicae* induces jasmonic acid (JA) and JA-inducible genes in local and systemic tissues of infected plants and this information is converted to an abscisic acid (ABA) response and the induction of ABA-responsive genes in neighboring plants via a *P. indica* hyphal network.

## Materials and methods

### Growth of the organisms, *A. brassicae* spore infection, co-cultivation of arabidopsis and fungi

**Figure 1:**
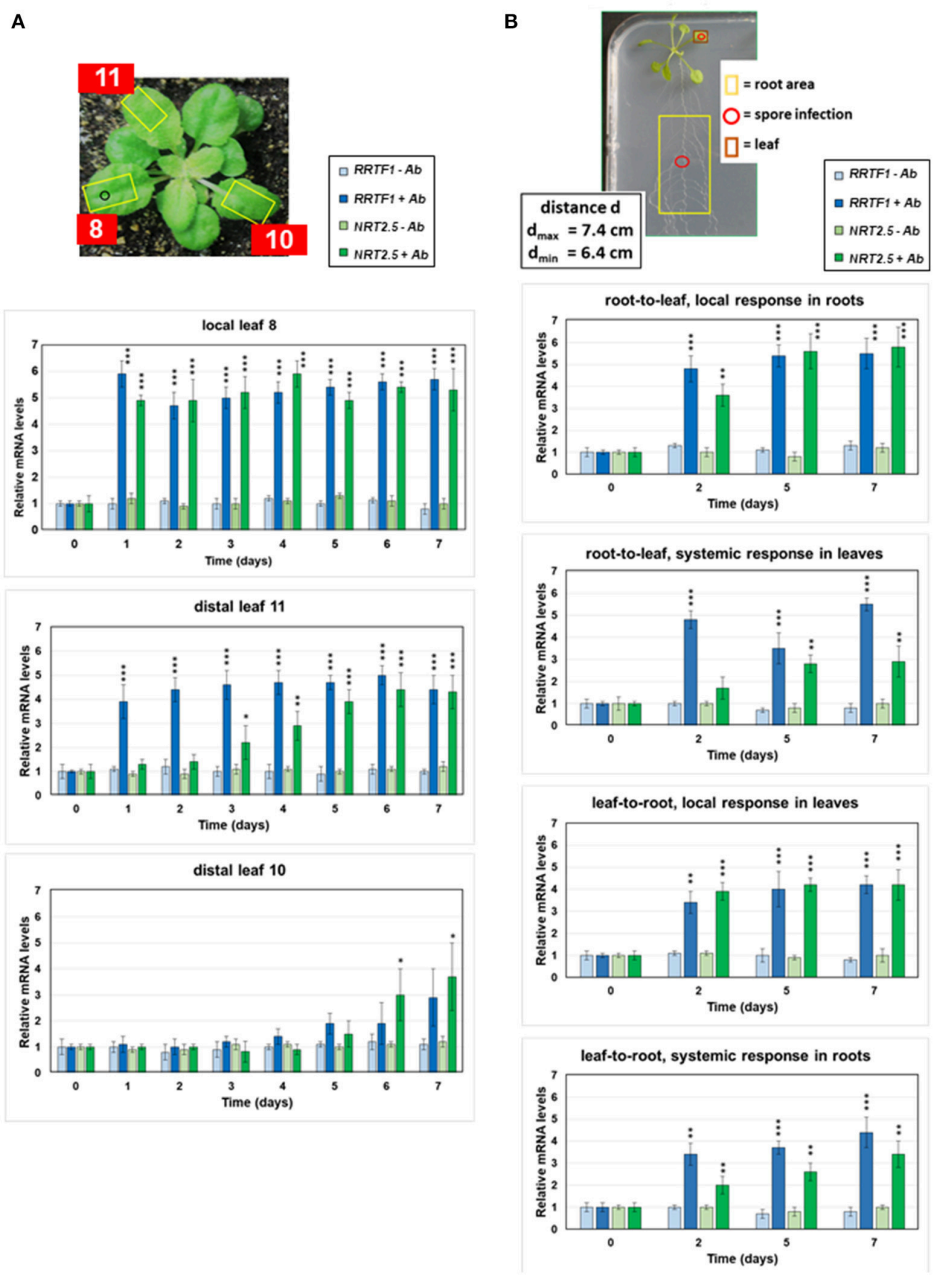
Local and systemic induction of *RRTF1* (blue bars) and *NRT2.5* (green bars) mRNA levels by *A. brassicae* spore infection. **(A)** The picture shows the experimental set-up. The leaf numbers of 4-week-old seedlings, the *A. brassicae* infection area (o) and the sections used for RNA extraction (yellow squares) are shown. *RRTF1* and *NRT2.5* mRNA levels in the local infected leaf no. 8 and the distal leaves no. 11 and 10. At day 0, infection was performed with an *A. brassicae* spore suspension and the mRNA accumulation was followed in infected (darker bars) and mock (water)-treated (lighter bars) seedlings over a period of 7 days. **(B)** The picture shows 2-week-old Arabidopsis seedlings grown in square Petri dishes for the measurements of *RRTF1* and *NRT2.5* mRNA levels in local and systemic tissues. The spore infection occurred either at the leaf or at the root (red o). The local and systemic leaf areas harvested for RNA isolation are indicated (red and yellow squares). The graphs show *RRTF1* (blue bars) and *NRT2.5* (green bars) mRNA levels in local and systemic tissues 0, 2, 5, and 7 days after application of an *A. brassicae* spore suspension (darker bars), lighter bars show water controls. All RNA data are based on 6 independent experiments with 10 seedlings for each treatment. The mRNA levels for the 4 datasets at day 0 were set as 1.0 and all other values were expressed relative to them (±SEs). Asterisks indicate significant differences of the values for Alternaria-treated tissue compared to the corresponding water control at the same time point, as determined by Student's *t*-test (^*^P ≤ 0.1; ^**^P ≤ 0.01; ^***^P ≤ 0.001). ^***^ >, all higher values have *P* ≤ 0.001 compared to mock-treated controls.

For the analysis of radial systemic signaling, a leaf of a 4 week-old Arabidopsis plant grown on garden soil in a square pot (6 × 6 cm) under short day conditions was infected with 2 μl of an Alternaria spore suspension (1 × 10^6^ spores/ml), as shown in Figure 1A. Preparation of the spore suspension has been described in Michal Johnson et al. (2014). The infected local leaf (no. 8, cf. Farmer et al., 2013) and the non-infected distal leaves (no. 11, according to Dengler, 2006, and no. 10) were harvested at the time points indicated and immediately frozen in liquid nitrogen for RNA extraction.

For the analysis of axial systemic signaling, Arabidopsis seedlings were grown vertically on ½ MS medium in square plates for 2 weeks at long-day conditions (80 μmol m^−2^ s^−1^; Figure 1B). The root area or leaf marked in Figure 1B were infected with 2 μl of an Alternaria spore suspension (1 × 10^6^ spores/ml) and both tissues were harvested separately at the time points indicated in the figures, immediately frozen in liquid nitrogen, and used for RNA extraction.

For interplant communication assays, *P. indica* was cultured as described previously (Verma and Varma, 1998; Peškan-Berghöfer et al., 2004) in Petri dishes on a modified Kaefer's medium (KM). The plates were kept at room conditions for 2 weeks. A plaque of 5 mm diameter of KM media with and without (control) *P. indica* mycelium was transferred to the middle of a new plate, as shown in **Figure 3**. Four seedlings (2 week old, grown on ½ MS medium) were positioned on each plate as shown in **Figure 3**, so that the root tips were in contact with the fungal (or control) plaque. The plates were kept for 2 days in continuous light of 65 μmol m^−2^ s^−1^, before the onset of experimentation.

To inhibit the information flow from the leaves of the three seedlings infected with *A. brassicae* spores to the leaves of the unaffected neighboring seedling (cf. **Figure 3**, seedlings 5 and 6), three experiments were performed: (a) Roots of the infected and non-infected seedlings were separated by a cellophane membrane which prevents physical contact. (b) Mycelium and agar between the infected and uninfected seedlings were cut with a razor blade every 2nd day, starting at day 0. (c) 10 ppm benomyl which kills the mycelium (Paul et al., 2001) was applied to the hyphae connecting the seedlings, at day 0, 2, 5, and 7. For some experiments shown in **Figure 4**, *P. indica* was replaced by the fungi *Absidia glauca* or *Mucor mucedo* (obtained from Institute of General Microbiology and Microbe Genetics, Jena).

For the data shown in Table 1, 4 seedlings (2 week-old, grown on MS medium with 0.3% (w/v) gelrite) were transferred to PNM plates (Michal Johnson et al., 2014) with 0.3% (w/v) gelrite and *P. indica* (or a control plate without the fungus). The fungus was pre-grown on the plate for 7 days. Two of the seedlings (in alternate order) were infected with *A. brassicae* spores, and in the control plates, two were mock-treated with water. After 12 days of co-culture, the intact seedlings with their roots were removed from the plates, and those not treated with *A. brassicae* were used for RNA and hormone measurements.

**Table 1:** The experimental set-up is shown in Figures S1: 4 Arabidopsis seedlings were either transferred to plates without *P. indica* or to plates with a 1 week-old *P. indica* fungal lawn for 12 days.

**A**				
**Parameter measured in roots of *A. brassicae*-infected seedlings**	**No *P. indica* lawn No *A. brassicae* infection**	**No *P. indica* lawn *A. brassicae* infection**	***P. indica* lawn No *A. brassicae* infection**	***P. indica* lawn *A. brassicae* infection**
JA [ng/g dry weight]	1510 ± 127	12610 ± 333^***^	1480 ± 230	10010 ± 240^***^
JA-Ile [ng/g dry weight]	5.1 ± 1.2	19.2 ± 3.4^***^	3.3 ± 0.7	4.7 ± 2.5
*cis*-OPDA [ng/g dry weight]	805 ± 99	1553 ± 445^**^	561 ± 56	1403 ± 301^**^
SA [ng/g dry weight]	1216 ± 144	455 ± 51^**^	3420 ± 341^***^	2105 ± 167^***^
ABA [ng/g dry weight]	2.2 ± 0.4	3.5 ± 0.9	2.1 ± 0.3	3.5 ± 1.3^***^
**B**				
**Parameter measured in roots of seedlings not treated with** ***A. brassicae***	**No** ***P. indica*** **lawn No** ***A. brassicae*** **infection of neighboring seedlings**	**No** ***P. indica*** **lawn** ***A. brassicae*** **infection of neighboring seedlings**	***P. indica*** **lawn No** ***A. brassicae*** **infection of neighboring seedlings**	***P. indica*** **lawn** ***A. brassicae*** **infection of neighboring seedlings**
JA [ng/g dry weight]	**1510** ± **127**	1980 ± 109	**1480** ± **230**	4410 ± 640^***^
JA-Ile [ng/g dry weight]	**5.1** ± **1.2**	11.6 ± 3.4	**3.3** ± **0.7**	15.7 ± 5.4
*cis*-OPDA [ng/g dry weight]	**805** ± **99**	870 ± 127	**561** ± **56**	1227 ± 234^***^
SA [ng/g dry weight]	**1216** ± **144**	1010 ± 121	**3420** ± 341^***^	2105 ± 167^**^
ABA [ng/g dry weight]	**2.2** ± **0.4**	4.5 ± 0.6	**2.1** ± **0.3**	19.5 ± 4.6^***^
*NRT2.5* mRNA level	1.0 ± 0.3	1.1 ± 0.4	1.6 ± 0.3	3.5 ± 0.6^***^
*RRTF1* mRNA level	1.0 ± 0.4	1.5 ± 0.4	1.3 ± 01	3.7 ± 0.9^***^
*PDF1* mRNA level	1.0 ± 0.2	1.4 ± 0.5	0.8 ± 0.4	2.2 ± 0.3^**^
*VSP2* mRNA level	1.0 ± 0.2	1.3 ± 0.2	1.0 ± 0.2	1.9 ± 0.4^**^
*JAR1* mRNA level	1.0 ± 0.3	1.6 ± 0.2	0.9 ± 0.4	2.1 ± 0.5^*^
*PR1* mRNA level	1.0 ± 0.4	1.0 ± 0.3	5.4 ± 0.7^***^	1.9 ± 03
*RD29A* mRNA level	1.0 ± 0.2	1.0 ± 0.2	1.2 ± 0.2	3.3 ± 0.3^***^
*RAB18* mRNA level	1.0 ± 0.4	1.3 ± 0.2	1.3 ± 0.3	3.5 ± 0.6^***^
*JAM1* mRNA level	1.0 ± 0.3	1.3 ± 0.3	1.2 ± 0.3	4.4 ± 0.5^***^
**C**				
**Parameter measured in roots of** ***abi1-5*** **seedlings not treated with** ***A. brassicae*** (Finkelstein and Lynch, 2000)				
*RD29A* mRNA level	1.0 ± 0.3	1.4 ± 0.3	1.1 ± 0.1	0.7 ± 0.3
*RAB18* mRNA level	1.0 ± 0.1	1.2 ± 0.3	1.3 ± 0.4	1.0 ± 0.3
*JAM1* mRNA level	1.0 ± 0.3	1.1 ± 0.1	1.2 ± 0.3	1.1 ± 0.1
**D**				
**Parameter measured in roots of** ***aba2-1*** **seedlings not treated with** ***A. brassicae***				
*RD29A* mRNA level	1.0 ± 0.0	1.5 ± 0.1	13 ± 0.1	1.5 ± 0.2
*RAB18* mRNA level	1.0 ± 0.2	1.3 ± 0.1	1.3 ± 0.2	1.7 ± 0.4
*JAM1* mRNA level	1.0 ± 0.3	1.2 ± 0.2	1.2 ± 0.2	1.4 ± 04

Two of the four 4 seedlings were inoculated with an A. brassicae spore suspension applied to the leaves, or they were mock-treated with water, the other two seedlings remained untreated. After 12 days the treated **(A)** and untreated **(B)** seedlings were removed from the gelrite and the phytohormone (and mRNA levels) determined in their roots. **(C,D)**, same as **(B)**, except as the non-A-brassicae treated seedlings was abi5-1 or aba2-1. Based on 5 (hormone data) and 6 (RNA data) independent experiments, bars represent SEs. Asterisks indicate significant differences of the values compared to the corresponding control (no P. indica, no A. brassicae, column 1), as determined by Student's t-test (

* *P ≤ 0.1*;

** *P ≤ 0.01*;

*** *P ≤ 0.001)*.

*Light grew data are identical in **(A,B)***.

All Arabidopsis mutants used in this study have been described: *npr1* (Cao et al., 1997)*, jar1* (Staswick et al., 1992)*, myb72* (van der Ent et al., 2008), and *rbohD* knockout (Torres et al., 2002; gift from Prof. Jonathan DG Jones, Warwick, UK). The ABA mutants *abi5-1* and *aba2-1* were a gift from Prof. A. Gierl (Weihnstephan, Germany).

### RNA analysis

RNA was isolated from shoots and roots with an RNA isolation kit (RNeasy, Qiagen, Hilden, Germany). Reverse transcription of 1 μg of total RNA was performed with oligo dT Primer and the Omniscript RT Kit (Qiagen, Hilden, Germany). Real-time quantitative reverse transcription-PCR (RT-PCR) was conducted with the following primer pairs:

*NRT2.5* (At1G12940, CAGCTGATCATGCCCATCGTGTTC, GCGATGCATAAATCTGGAGAGAGGG), *RRTF1* (At4g34410, ACAGTGATAAGCGCGGGAAT, TCCACAAAGGGGAAGTTGAG), *JAM1* (At2g46510, CTCCTCGGCCACGATGTCTCTCCGC, CATAATCCGCCAAAATCTCTTCCATTCCTTC), *RD29A* (At5G52310, GGTTGAAGAAGATGATGATG, GGAAGACACGACAGGAAA), *RAB18* (At5g66400, ATTCCCTTCTTCCTCCTC, TGAAGGCTTTGGAACTGG), housekeeping gene *GAPDH* (At3g04120, GAGCTGACTACGTTGTTGAG, GGA GACAATGTCAAGGTCGG).

The primer pairs used for *PDF1.2, VSP2*, and *JAR1* were described in Scholz et al. (2014). Quantification of *A. brassicae* in infected and non-infected plant tissue (Figure **3C**) was performed with the *AbreATr1* gene marker (Guillemette et al., 2004, ACCCGCATTCCTCGCCAAA, AAGTCAAGGATTGTGTCGAGCTT) as described in Michal Johnson et al. (2014).

RT-PCR was performed using the Bio-Rad CFX connect real-time system and Bio-Rad CFX manager version 3.1 (Bio-Rad, Munich, Germany). For the amplification of the PCR products, Eva green (Bio-Rad) and Dream Taq DNA polymerase were used in a final volume of 20 μl. The CFX real-time PCR was programmed to 95°C 2 min, 39 × (95°C 30 s, 60°C 40 s, 72°C 45 s), 72°C 8 min followed by a melting curve program (55–95°C in increasing steps of 0.5°C). Annealing temperature was calculated for each primer pair. All reactions were repeated three times. The mRNA levels for each cDNA probe were normalized with respect to the *GAPDH* mRNA levels.

### Phytohormone measurement

Leaf and root material was frozen in liquid nitrogen and kept at −80°C until use. Thirty to One hundred and thirty milligrams of leaf or root material was ground with mortar and pestle, and extracted with 1.2 ml of methanol containing 24 ng of 9,10-D_2_-9,10-dihydrojasmonic acid, 24 ng D_4_-salicylic acid (Sigma-Aldrich), 24 ng D_6_-abscisic acid (Santa Cruz Biotechnology, Santa Cruz, U.S.A.), and 4.8 ng of JA-^13^C_6_-Ile conjugate as internal standards. JA-^13^C_6_-Ile conjugate was synthesized as described by Kramell et al. (1988) using ^13^C_6_-Ile (Sigma-Aldrich). The homogenate was mixed for 30 min and centrifuged at 14,000 rpm for 20 min at 4°C. The supernatant was collected. The homogenate was re-extracted with 500 μl methanol, mixed well, centrifuged and supernatants were pooled. The combined extracts were evaporated in a speed-vac at 30°C and re-dissolved in 250 μl methanol. Chromatography was performed on an Agilent 1200 HPLC system (Agilent Technologies). Separation was achieved on a Zorbax Eclipse XDB-C18 column (50 × 4.6 mm, 1.8 μm, Agilent). Formic acid (0.05%) in water and acetonitrile were employed as mobile phases A and B, respectively. The elution profile was: 0–0.5 min, 5% B; 0.5–9.5 min, 5–42% B; 9.5–9.51 min 42–100% B; 9.51–12 min 100% B, and 12.1–15 min 5% B. The mobile phase flow rate was 1.1 ml/min. The column temperature was maintained at 25°C. An API5000 tandem mass spectrometer (Applied Biosystems) equipped with a Turbospray ion source was operated in negative ionization mode. The instrument parameters were optimized by infusion experiments with pure standards, where available. The ionspray voltage was maintained at −4,500 eV. The turbo gas temperature was set at 700°C. Nebulizing gas was set at 60 psi, curtain gas at 25 psi, heating gas at 60 psi, and collision gas at 7 psi. Multiple reaction monitoring (MRM) was used to monitor analyte parent ion → product ion: m/z 136.9 → 93.0 [collision energy (CE) −22 V; declustering potential (DP) −35 V] for SA; m/z 140.9 → 97.0 (CE −22 V; DP −35 V) for D4-SA; m/z 209.1 → 59.0 (CE −24 V; DP −35 V) for JA; m/z 213.1 → 56.0 (CE −24 V; DP −35 V) for 9,10-D2-9,10-dihydrojasmonic acid; m/z 263.0 → 153.2 (CE −22 V; DP −35 V) for ABA; m/z 269.0 → 159.2 (CE −22 V; DP −35 V) for D6-ABA; m/z 322.2 → 130.1 (CE −30 V; DP −50 V) for JA-Ile conjugate; m/z 328.2 → 136.1 (CE −30 V; DP −50 V) for JA-^13^C_6_-Ile conjugate. Both Q1 and Q3 quadrupoles were maintained at unit resolution. Analyst 1.5 software (Applied Biosystems) was used for data acquisition and processing. Linearity in ionization efficiencies were verified by analyzing dilution series of standard mixtures. Phytohormones were quantified relative to the signal of their corresponding internal standard. For quantification of 12-oxophytodienoic acid, *cis*-OPDA, 9,10-D_2_-9,10-dihydro-JA was used as the internal standard applying an experimentally determined response factor of 1.

### Confocal microscopy

**Figure 2:**
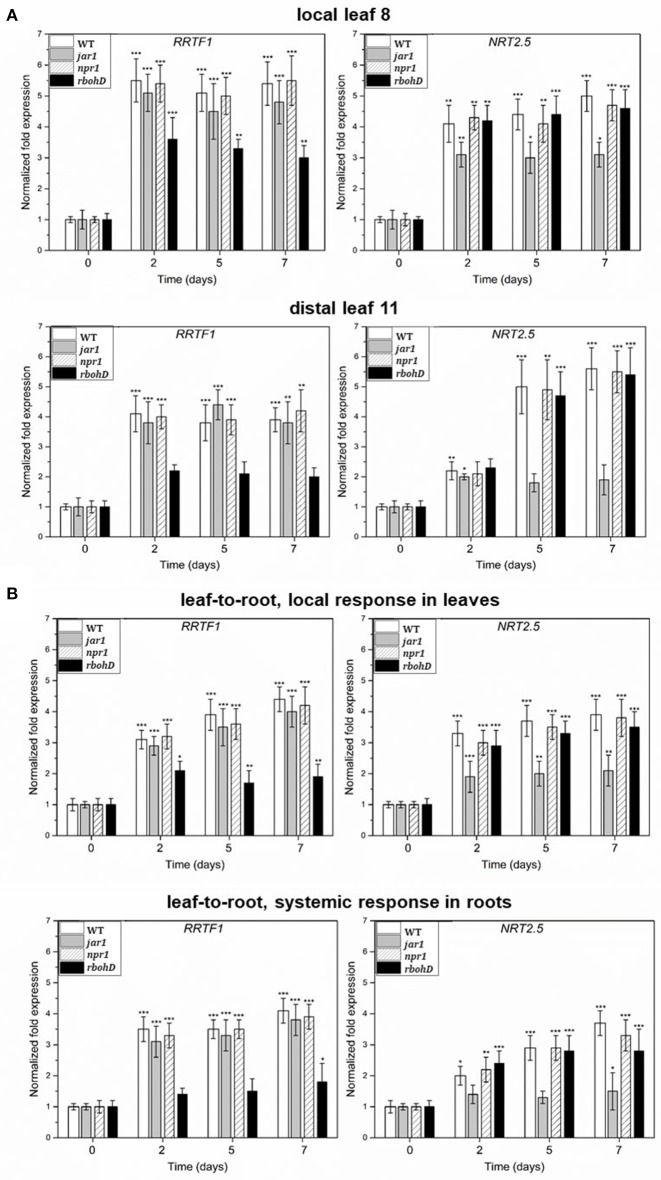
**(A)** Induction of *RRTF1* and *NRT2.5* mRNA levels in the *A. brassicae*-infected leaf no. 8 and the systemic leaf no. 11 of WT, *jar1, npr1*, and *rbohD* plants. The treatment was the same as described in the legend to Figure 1A. **(B)** Induction of *RRTF1* and *NRT2.5* mRNA levels in the *A. brassicae*-infected leaves and the non-infected roots of WT, *jar1, npr1*, and *rbohD* seedlings on agar plates, as shown in the Figure 1B. The treatment was the same as described in the legend to Figures 1A,B. The mRNA levels at day 0 were set as 1.0 and all other values were expressed relative to them (±SEs). Asterisks indicate significant differences of the values compared to the mock-treated controls (which are not shown, but comparable to the results shown for WT material in Figure 1), as determined by Student's *t*-test (^*^P ≤ 0.1; ^**^P ≤ 0.01; ^***^P ≤ 0.001).

Twelve days after infection of *A. thaliana* with GFP-labeled *P. indica* (gift from Prof. P. Schäfer, Warwick, UK) root colonization was imaged using a LSM 880 (Zeiss Microscopy GmbH, Jena, Germany) with the 488 nm laser line of an argon multiline laser (Figure 2B). Images were taken with a 40x objective (Plan-Apochromat 40x/0.8). A maximum intensity projection was performed from a z-stack of 26 plains each 0.5 μm. Digital images were processed by ZEN software.

### ROS measurements

Quantitative ROS measurements from leaves were performed with the Amplex Red hydrogen peroxide/peroxidase assay kit (Molecular Probes, Invitrogen, Carlsbad, CA, USA) according to the manufacturer's instructions (https://tools.thermofisher.com/content/sfs/manuals/mp22188.pdf). Leaf sections of 0.5–1 mm width were incubated in the reaction mixture for 10 min in dark at room temperature. The fluorescence intensity was quantified with a fluorescence microplate reader (TECAN Infinite 200 plate reader; Crailsheim, Germany) with excitation at 540 nm and emission at 610 nm. H_2_O_2_ was used to prepare the standard curve. The reaction mixture without the molecular probe or without the plant material served as control.

## Results

### Radial and axial systemic induction of *NRT2.5* and *RRTF1* by *Alternaria brassicae* infection

Since interplant signal transfer requires systemic information flow within a plant, we looked for genes which responded systemically to various threats in preliminary experiments and decided for *NRT2.5* and *RRTF1* as read-out to assay information transfer for radially and axially traveling signals. The genes were chosen for study because they responded to various disease-inducing fungal infections in preliminary studies, are not directly related to phytohormone responses, represent responses to systemic signals which are not directly related to each other, and are involved in the primary metabolism and defense strategies (cf. section Introduction).

Arabidopsis leaf no. 8 was infected with 2 μl of an Alternaria spore suspension (1 × 10^6^ spores/ml) or mock-treated with water. The *NRT2.5* and *RRTF1* mRNA levels were measured in the infected leaf and in distal non-infected leaf no. 11, which is vascularly connected to the infected leaf (Figure 1A, Dengler, 2006). A strong increase in the mRNA levels relative to the mock-treated controls was detectable in the infected leaf 1 day after spore application and the mRNA levels remained high until the 7th day. In distal leaf no. 11, a significant increase was detected between 1 (*RRTF1*) and 3 (*NRT2.5*) days after infection and the *RRTF1* mRNA level responded much earlier than the *NRT2.5* mRNA level (Figure 1A). Since only a low elevation in the *NRT2.5* and *RRTF1* mRNA levels was observed in the non-connected distal leaf no. 10, and the response started much later (Dengler, 2006; Kiep et al., 2015), a volatile compound as signal transducer is unlikely (Figure 1A). Likewise, when the roots of seedlings were infected with *A. brassicae* spores (Figure 1B), the expression of the two genes increased within the first 2 days in the roots. An increased expression in the leaves was detected between the 2nd (*RRTF1*) and 5th (*NRT2.5*) day (Figure 1B). Again, the mRNA level for *RRTF1* responded earlier than that for *NRT2.5*. The information also traveled from the leaf toward the root with a comparable induction observed in roots when the leaves were infected with *A. brassicae* spores (Figure 1B). This indicates that *NRT2.5* and *RRTF1* are systemically induced in non-infected leaves or roots by radially and axially migrating signals, and the axial information flow is bidirectional.

### Local and systemic induction of *NRT2.5* is JAR1-dependent and induction of *RRTF1* is RBOHD-dependent

*A. brassicae* infection induces JA, but not salicylic acid (SA) accumulation in the infected host tissue (Michal Johnson et al., 2014). To test whether *RRTF1* and *NRT2.5* regulation is linked to these hormones, we measured the induction of the RNA levels in the hormone mutants *jar1* and *npr1*. JAR1 catalyzes the formation of the biologically active jasmonyl-isoleucine (JA-Ile) conjugate, and NPR1 is a receptor for SA (Wu et al., 2012). The mRNA levels were determined in infected local leaves (as shown in Figures 1A,B), the distal leaf no. 11 (as shown in Figure 1A) and roots of seedlings with leaf infection (shown in Figure 1B). *RRTF1* expression was comparable to the WT in *jar1* and *npr1* plants. *NRT2.5* induction in local (leaf no. 8) and distal (leaf no. 11) tissue was inhibited in the *jar1* mutant, but not in the *npr1* mutant in which part of the SA response was inhibited (cf. Herrera-Vásquez et al., 2015; Figure 2). Furthermore, *RRTF1* expression has been shown to be induced by ROS (Khandelwal et al., 2008; Matsuo et al., 2015) and *A. brassicae* infection reported to stimulate ROS accumulation in local and systemic tissues, mainly via the H_2_O_2_-producing RBOHD, a plasmamembrane-localized NADH oxidase preferentially activated in response to pathogen attacks (Michal Johnson et al., 2014; Matsuo et al., 2015 and ref. therein;). The stimulatory effect on *RRTF1* expression in both local and systemic tissues was reduced in the *rbohD* mutant (Figure 2), while *NRT2.5* expression was not affected. This suggests the involvement of ROS produced by RBOHD in the local and systemic *RRTF1* response. Finally, MYB72, a transcription factor up-regulated by infection of non-pathogenic rhizobacteria that plays a crucial role in induced systemic resistance and root-to-shoot signaling (Segarra et al., 2012), was found to be not involved in the axial systemic information flow from the roots to the leaf leading to *NRT2.5* and *RRTF1* induction, since this information flow was not impaired in the *myb72* mutant (data not shown, since they are not significantly different from the WT responses). Taken together, local and systemic induction of *NRT2.5*, but not *RRTF1*, is partially dependent on jasmonate signaling, whereas local and systemic *RRTF1* regulation is partially dependent on RBOHD.

### *Piriformospora indica* involvement in interplant communication: experimental set-up

**Figure 3:**
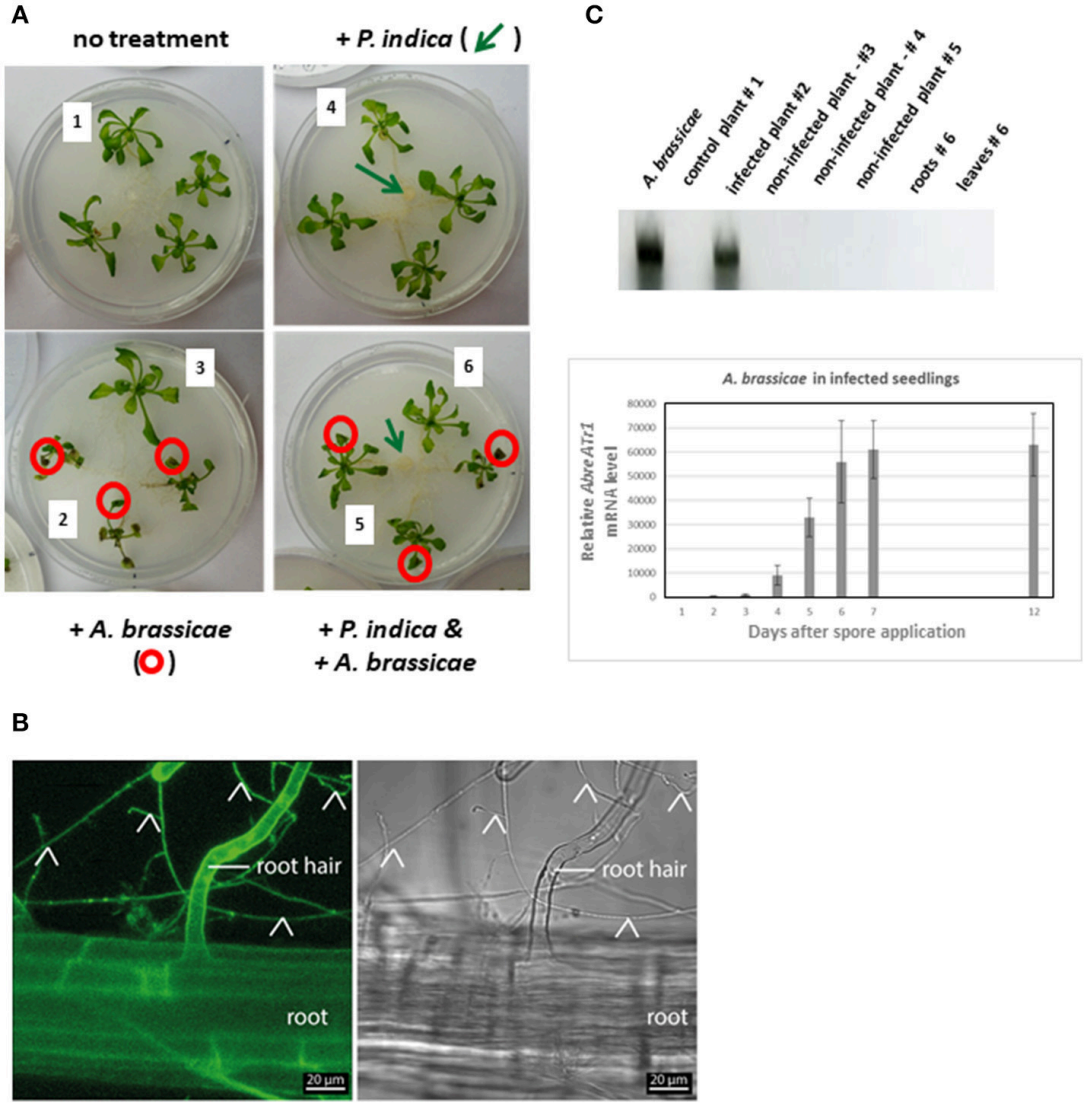
**(A)** Experimental set-up for interplant communication. Four 2-week-old Arabidopsis seedlings were positioned in a fresh Petri dish. The roots were not connected (left two Petri dishes) or connected (right two Petri dishes) to each other *via P. indica* hyphae; *P. indica* was inoculated 1 week before transfer of the seedlings to the plates (cf. section Methods and Materials). The leaves of six seedlings were inoculated with an *A. brassicae* spore suspension (circled in red). The leaves of the seedlings 1–6 were harvested 0, 2, 5, 7, and 12 days after infection for RNA extraction and qPCR analyses. (1, no treatment; 2, *A. brassicae* infected material; 3, seedlings grown next to *A. brassicae*-infected seedlings, 4–6, as 1–3, except that seedlings were connected by a *P. indica* hyphal network) **(B)** Confocal image of an Arabidopsis root grown on the *P. indica* hyphal lawn for 12 days (end of experiment). The signal detected with the GFP channel is shown on the left and a bright field image on the right; root hairs are indicated by the label and fungal hyphae by arrow heads. **(C)** Quantification of *A. brassicae AbreATr1* mRNA by qPCR. The gel shows amplified cDNA fragments from mRNAs of the seedlings #1–6 after 12 days of co-cultivation which are shown in panel A. *A. brassicae*: PCR product from RNA of an *A. brassicae* culture was used as positive control. The graph shows relative *AbreATr1* mRNA levels of the infected seedlings #2 between 0 and 12 days after spore application. Based on 6 independent experiments with 10 seedlings each. Error bars are SEs.

Figure 3A shows the experimental design used for interplant communication assays. Four Arabidopsis seedlings were grown in a Petri dish. In plate 1, the four seedlings were not exposed to any fungus (sample 1), in plate 2, the leaves of three seedlings were infected with an *A. brassicae* spore suspension (sample 2), while the fourth seedling remained uninfected (sample 3). In the 3rd and 4th plate, the Arabidopsis seedlings were grown in the presence of a *P. indica* hyphal network. While seedlings in the 3rd plate received no additional treatment (sample 4), the leaves of three seedlings in the 4th plate were infected with *A. brassicae* spores (sample 5). The 4th seedling was not infected by the pathogen, but connected to the infected seedlings via a *P. indica* hyphal network (sample 6). *P. indica* forms a hyphal network that connects the roots of the 4 plants on the plate (Figure 3B). The majority of the hyphae associated with the seedlings can be detected around the primary, secondary and lateral roots. The percentage of hyphae that are visible inside root cells was low. Until the end of the experiment (12 days after infection), we could not detect dead plant cells caused by *P. indica* infection (cf. Figure 3B). *A. brassicae* spores germinated on the infected seedling but no pathogen DNA was detected by real-time PCR in the non-infected seedlings growing next to the infected seedlings (Figure 3C). Time course experiments demonstrated that significant amounts of pathogen DNA were first detected 48 h after spore application in the infected seedlings. As expected, between the third and 12th day, the amount of pathogen DNA in the infected seedlings increased dramatically (Figure 3C).

### *NRT2.5* and *RRTF1* respond to signals from *A. brassicae*-treated neighboring plants through a *P. indica* network

**Figure 4:**
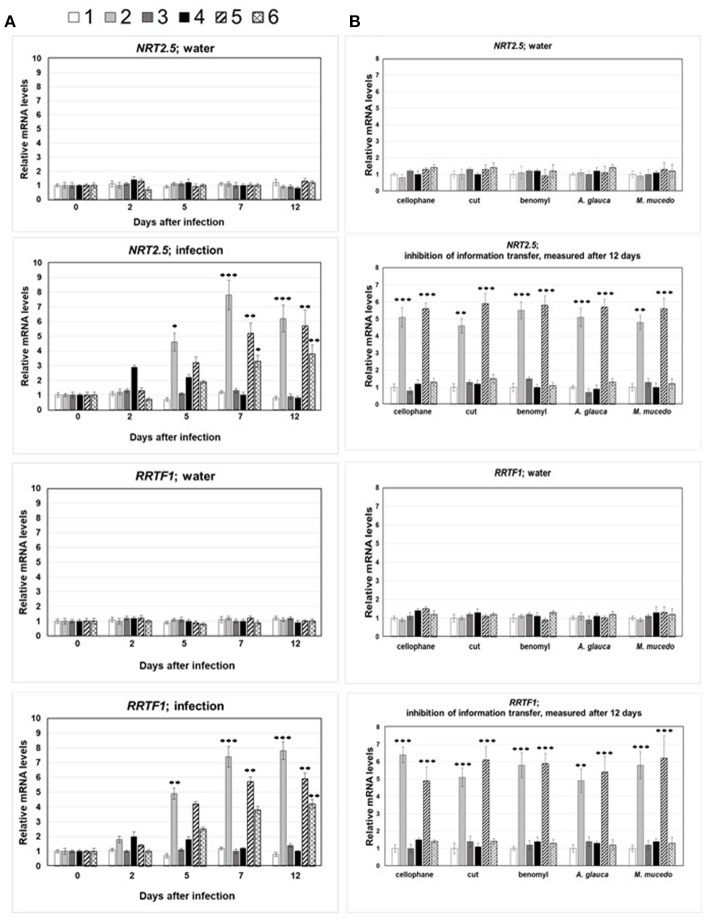
**(A)** *NRT2.5* and *RRTF1* mRNA levels in infected and systemic leaves following *A. brassicae* spore application at day 0. The seedlings were either mock-treated (water) or inoculated with *A. brassicae* spores (infection). The numbers 1–6 refer to the seedlings shown in Figure 3A: 1, no treatment; 2, *A. brassicae* infected material; 3, seedlings grown next to *A. brassicae*-infected seedlings, 4–6, as 1–3, except that seedlings were connected by a *P. indica* hyphal network. The mRNA levels at the time point of infection (*t* = 0) was set as 1.0 and all other values are expressed relative to them. **(B)** *NRT2.5* and *RRTF1* mRNA levels in leaves of neighboring non-infected plants requires *P. indica* hyphal connection. Same experiment as in **(A)**, but the connection between the roots via *P. indica* hyphae were interrupted by the insertion of a cellophane membrane (cellophane), or the hyphal connections were cut with a razor blade every 2nd day (cut), or *P. indica* were treated with benomyl at day 0, 2, 5, and 7 (benomyl). *A. glauca, M. mucedo*; *P. indica* was replaced by these fungi. All measurements were performed 12 dai and are based on 6 independent experiments with 10 plants each. Asterisks indicate significant differences of the values for *A. brassicae*-treated tissue compared to the corresponding water control at the same time point, as determined by Student's *t*-test (^*^P ≤ 0.1; ^**^P ≤ 0.01; ^***^ *P* ≤ 0.001). The data for the water control did not change significantly within the 12 days and were below 1.5 ± 0.4.

Figure 4A demonstrates that *NRT2.5* and *RRTF1* expression is also induced by signals from neighboring plants. The mRNA abundance of these two genes increased in the leaves of non-infected seedlings when they were connected by a *P. indica* mycelial network to the infected seedlings (Figure 4A). No induction was detected when the *P. indica* hyphal connection was interrupted, either by the insertion of a cellophane membrane which cannot be penetrated by hyphae (Vahabi et al., 2015a,b), by cutting the hyphae with a razor blade (cut) or application of 10 ppm of benomyl to the *P. inidca* hyphae, which kills the mycelium (Paul et al., 2001; Figure 4B). Interestingly, *P. indica* could not be replaced by *Absidia glauca* or *Mucor mucedo* (Figure 4B), two fungi which associate with but do not colonize Arabidopsis roots. This suggests that a physical contact *via* the *P. indica* hyphal network is required for *NRT2.5* and *RRTF1* induction in neighboring non-stress exposed plants.

Furthermore, induction of *NRT2.5* and *RRTF1* mRNA was measured for the *jar1* and *rbohD* mutants. Using the same experimental design as shown in Figure 3, wild-type seedlings were replaced by either *jar1* or *rbohD* seedlings, in all possible combinations. Consistent with the results obtained for systemic signal propagation within the infected plant, we observed that if one of the partners was *jar1* or *rbohD*, there was no significant response of *NRT2.5* (*jar1*) or *RRTF1* (*rbohD*) in the leaves of the receiving plants connected via the *P. indica* hyphal connection to the infected plants (data not shown). It appears that the information cannot travel if one or both of the partners is a mutant, or occurs at a rate too low to measure.

### Volatiles do not induce *NRT2.5* and *RRTF1* expression in uninfected neighboring plants

**Figure 5:**
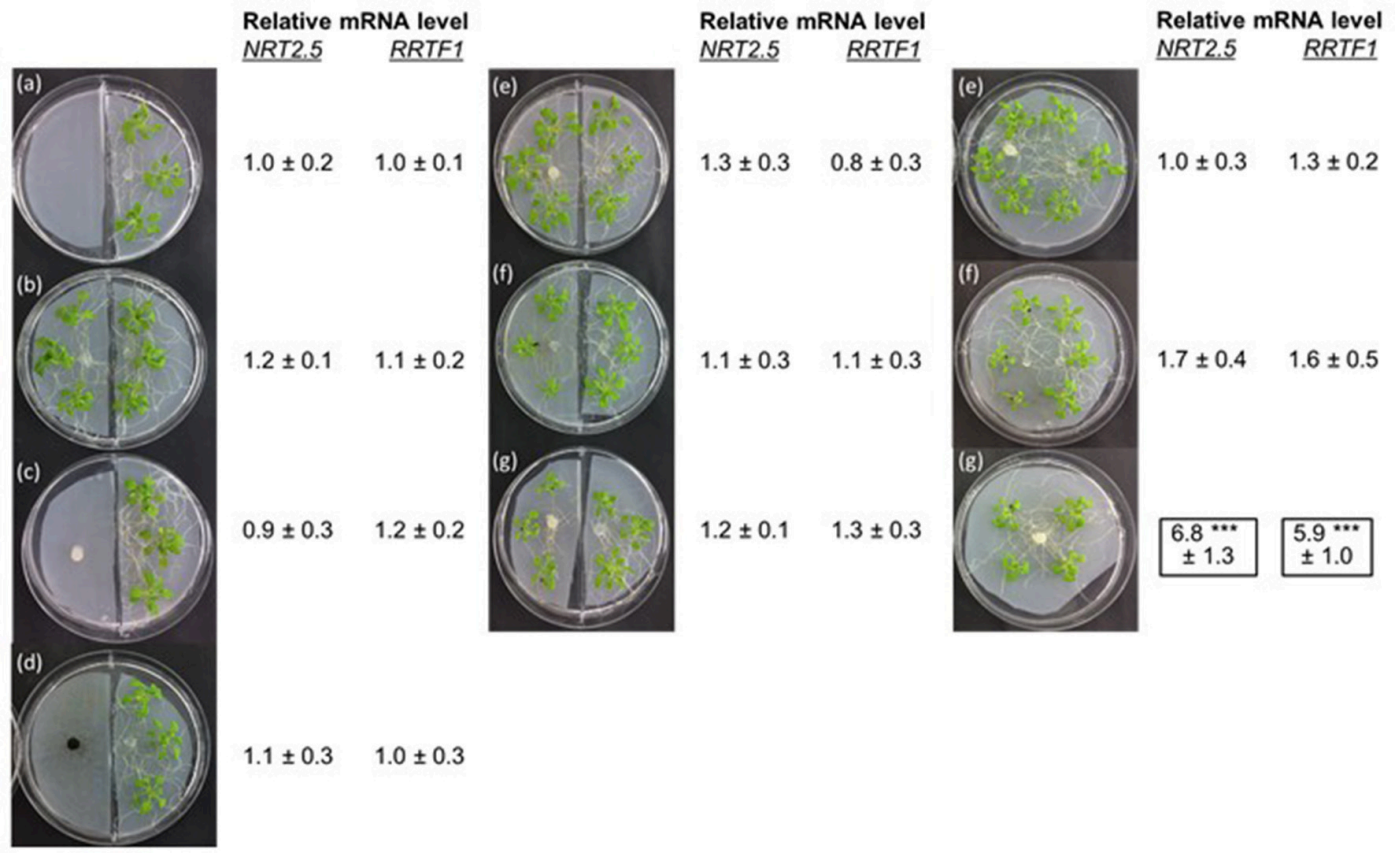
*NRT2.5* and *RRTF1* mRNA levels in Arabidopsis seedlings grown in the right chamber of a split Petri dish. The other chamber contained either PNM medium alone (a), or PNM medium with Arabidopsis seedlings (b), *P. indica* (c), or *A. brassicae* (d) hyphae or Arabidopsis seedlings co-cultivated with the fungi, as described in the legends to the Figures 2, 3 (e, *P. indica* alone, f, Arabidopsis infected with *A. brassicae* spores, g, Arabidopsis seedlings exposed to both fungi). The organisms grown in the three Petri dishes on the right site were not separated from each other. RNA extraction was performed 12 days. The mRNA levels of Arabidopsis seedlings with only PNM medium in the neighboring chamber was set as 1.0 and all other values are expressed relative to them. Based on 6 independent experiments with 10 seedlings for each treatment.

To confirm that volatiles do not participate in the stimulation of *NRT2.5* and *RRTF1* in uninfected seedlings, a split Petri dish experiment was performed. Wild-type Arabidopsis seedlings were grown on one half of the Petri dish, while in the other half, we grew either an Arabidopsis plant alone, one colonized by *P. indica*, one exposed to *A. brassicae*, or one with both *P. indica* and *A. brassicae*. Furthermore, Arabidopsis seedlings were exposed to *P. indica* or *A. brassicae* hyphae or a combination of both fungi in the neighboring chamber. In none of these experiments, did we observe elevated *NRT2.5* and *RRTF1* mRNA levels in Arabidopsis seedlings compared to the control (Figure 5). Stimulation of both mRNA levels were only measured in the experimental set-up with a *P. indica* hyphal connection between the two seedlings (marked in black in Figure 5). This supports the idea that a physical contact is required for the information transfer to the neighboring plant for the induction of *NRT2.5* and *RRTF1*.

### Phytohormone and phytohormone-responsive genes in infected and non-infected neighboring plants

**Figure 6:**
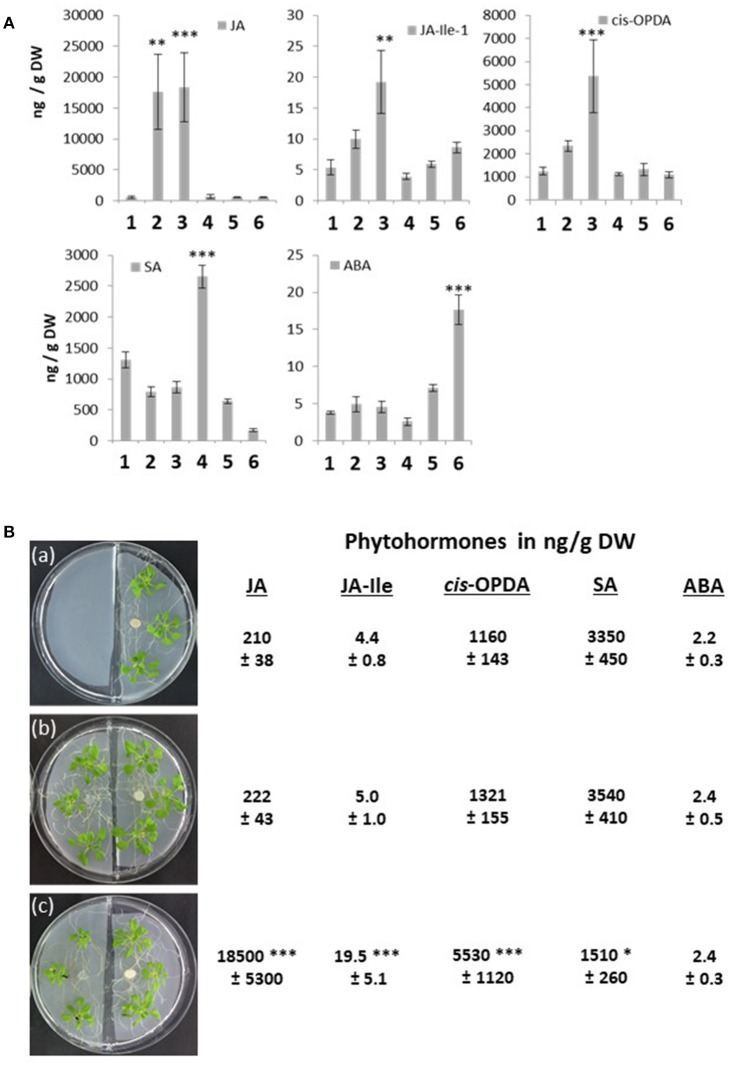
**(A)** Phytohormone concentrations in ng/g dry weight (DW) in the 6 seedlings (number 1–6) shown in Figure 3A. 1, no treatment; 2, *A. brassicae* infected material; 3, seedlings grown next to *A. brassicae*-infected seedlings, 4–6, as 1–3, except that seedlings were connected by a *P. indica* hyphal network. The experimental set-up is shown in Figure 3A. Based on 5 independent experiments, errors represent SEs. Asterisks indicate significant differences to the values for the untreated seedling number 1, by Student's *t*-test (^**^P ≤ 0,01; ^***^P ≤ 0.001). **(B)** Split Petri dish experiment with Arabidopsis seedlings. Left chamber: **(a)** empty, **(b)** Arabidopsis seedlings, **(c)** Arabidopsis seedlings infected with *A. brassicae* spores for 12 days. The hormone levels were determined for the seedlings grown on the right site in the Petri dish, which were grown in the presence of *P. indica* for 12 days. Based on 5 independent experiments, errors represent SEs. Asterisks indicate significant differences to the values of experiment **(a)**, by Student's *t*-test (^*^P ≤ 0.1, ^***^P ≤ 0.001).

To further elucidate how information about infection is transferred to non-infected neighboring plants, we checked defense-related phytohormone levels. *A. brassicae* is known to stimulate JA but not SA accumulation, whereas *P. indica* stimulates SA, but not JA accumulation (Michal Johnson et al., 2014). Consistent with these observations, plants which were only infected by *A. brassicae* (seedling 2 in Figure 3A) had higher JA levels compared to the untreated control (seedling 1 in Figure 3A), while the SA level was slightly reduced (Figure 6A). In seedlings which were only exposed to *P. indica* (seedling 4 in Figure 3A), the SA level was high, while the JA, JA-Ile and *cis*-OPDA levels were comparable to those in seedlings not exposed to any fungus (Figure 6A). Interestingly, *A. brassicae* infection also stimulated the JA, JA-Ile, and *cis*-OPDA levels in non-infected seedlings growing next to infected seedlings, if they were not connected via a *P. indica* hyphal network (seedling 3 in Figures 3A, 6A). This is particularly striking for *cis*-OPDA, the precursor for JA, and JA-Ile, the active form of JA (Figure 6A). Apparently, the information is either transferred through the gas phase or by chemical mediators diffusing through the agar to the non-infected neighboring plants. The JA, JA-Ile, and *cis*-OPDA levels were also upregulated in Arabidopsis plants that were growing separately from *A. brassicae*-infected plants in split Petri dishes (Figure 6B). The phytohormone levels in seedlings grown in split Petri dishes and those grown in the “normal” Petri dishes were comparable and not significantly different from each other (Figures 6A,B). This supports the idea of information transfer through the air. *A. brassicae*-induced JA accumulation was completely prevented and JA-Ile and *cis*-OPDA strongly reduced in infected (seedling 5 in Figure 3A) and neighboring (seedling 6 in Figure 3A) seedlings that were exposed to *P. indica*. This is most likely caused by the stimulating effect of *P. indica* on SA accumulation: its level is high in all seedlings which were growing on plates with *P. indica* but strongly inhibited when *A. brassicae* was present in the plate, either alone or in combination with *P. indica* (Figure 6). Thus, *P. indica* represses *A. brassicae*-induced JA, JA-Ile and *cis*-OPDA accumulation and *A. brassicae* represses *P. indica*-induced SA accumulation in both infected and non-infected plants. This is not surprising considering the crosstalk between the two hormones (cf. Caarls et al., 2015). Interestingly, in seedlings not infected by *A. brassicae*, but connected to infected seedlings via a *P. indica* hyphal network (seedling 6 in Figure 3A), the ABA level was > 3-fold higher. The ABA level was not stimulated by *P. indica* or *A. brassicae* alone, or the combination of both fungi, or in non-infected seedlings without *P. indica* fungal connection to infected seedlings (Figure 6). Moreover, the ABA level was not elevated in non-infected seedlings grown in the presence of *P. indica*, when *A. brassicae* infected seedlings were growing next to them in split Petri dishes, although they had elevated JA, JA-Ile, and *cis*-OPDA levels (Figure 6B). Thus, stimulation of ABA accumulation in non-infected seedlings growing next to *A. brassicae*-infected seedlings requires the *P. indica* hyphal bridge, and is apparently not caused by a JA-ABA crosstalk during the interactions (discussed in various contexts in Robert-Seilaniantz et al., 2011; Kazan and Manners, 2012; Yang et al., 2013; de Ollas and Dodd, 2016; Di et al., 2016; Verma et al., 2016).

**Figure 7:**
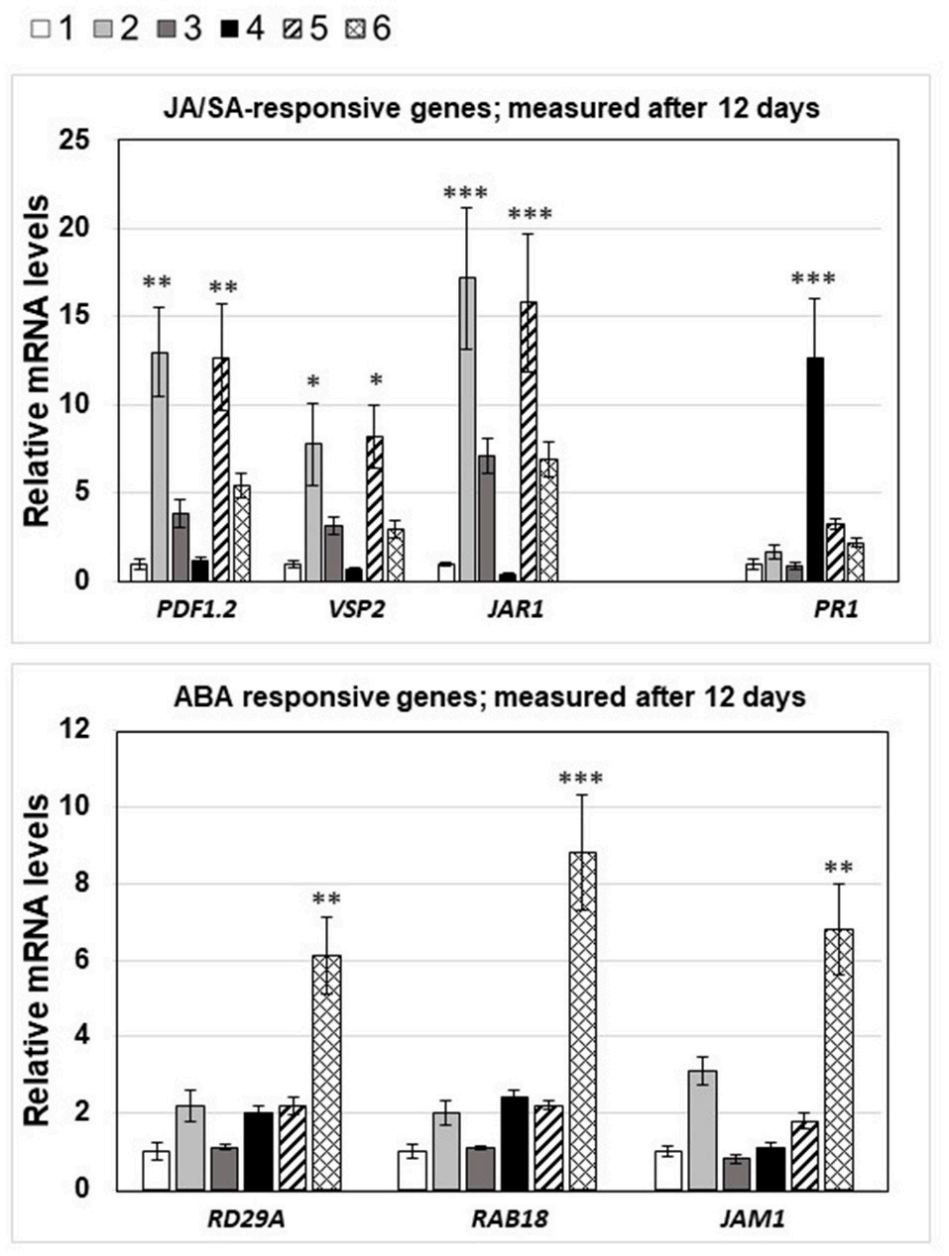
mRNA levels of JA-, SA-, and ABA-responsive genes in the 6 seedlings shown in Figure 3A, 12 days after spore infection: 1, no treatment; 2, *A. brassicae* infected material; 3, seedlings grown next to *A. brassicae*-infected seedlings, 4–6, as 1–3, except that seedlings were connected by a *P. indica* hyphal network. The experimental conditions were the same as described in the legend to Figure 4. Asterisks indicate significant differences of the values for Alternaria-treated tissue compared to the corresponding control (1, no treatment) at the same time point, as determined by Student's *t*-test (^*^P ≤ 0.1; ^**^P ≤ 0.01; ^***^P ≤ 0.001).

The different phytohormone levels are reflected in the expression pattern of phytohormone-responsive genes. Whenever JA, JA-Ile, or *cis*-OPDA was high, we observed elevated mRNA levels for the marker genes *PDF1.2, VSP2*, and *JAR1*, and elevated SA levels stimulated *PR-1* mRNA accumulation. Finally, the *RD29A, RAB18*, and *JAM1* mRNA levels were only up-regulated in seedlings with elevated ABA levels (Figure 7).

### Phytohormone levels and phytohormone-response genes in roots of infected and non-infected neighboring plants

Arabidopsis seedlings were grown on plates with low gelrite concentration either in the presence or absence of *P. indica* (Figure S1). Half of the seedlings were infected with the *A. brassicae* spore suspension (or water as control, marked with a red circle in Figure S1), while the other half remained untreated. After 12 days, the treated (Table 1A) untreated (Tables 1B–D) seedlings were removed for the plates, the roots were separated from the seedling and used for hormone or RNA analyses. From the comparison of the hormone levels shown in Tables 1A,B and those for the different treatments shown in Figure 1B, it is obvious that JA, JA-Ile, and *cis*-OPDA levels were stimulated by signals traveling through the *P. indica* hyphal network from *A. brassicae*-infected seedlings to the roots of the non-infected seedlings. The SA level was higher in roots exposed to *P. indica*, and partially repressed when *A. brassicae*-infected seedlings were connected to infected seedlings via the *P. indica* hyphae. Again, ABA was only upregulated in the roots of non-infected seedlings that were connected to infected seedlings via the *P. indica* hyphal network. This again excludes an information transfer through the gas phase and confirms the requirement of the *P. indica* hyphal network for ABA induction (cf. section Discussion).

Next, we checked the *NRT2.5* and *RRTF1* mRNA levels in the roots under the four different conditions and compared them to those for the JA-responsive genes *PDF1.2, VSP2*, and *JAR1*, the SA-inducible gene *PR-1* and the ABA-responsive genes *RD29A, RAB18*, and *JAM1* (Table 1B). Consistent with the results from Figure 5, the *NRT2.5* and *RRTF1* mRNA levels were up-regulated in the roots of non-infected seedlings when they were connected to infected seedlings *via* a *P. indica* hyphal network. This confirms that physical contact via the *P. indica* hyphal network is required for their induction. As shown previously (Kechid et al., 2013), beneficial root-colonizing microbes can induce *NRT2.5* expression and a small but not significant stimulation was detectable in the roots of *P. indica*-exposed seedlings (Table 1B). The JA-responsive genes were significantly stimulated in the roots of non-infected seedlings, when they were connected to infected seedlings via a hyphal network. In contrast to the results for the entire seedlings (Figure 6), an information transfer through the air or by chemical mediators in the medium does not play an important role. *P. indica* also stimulated the accumulation of SA and the expression of *PR1*, and this was inhibited when the seedlings were connected to an *A. brassicae*-infected neighboring plant. The ABA-inducible genes were only up-regulated in the roots of non-infected seedlings connected to the infected seedlings via a *P. indica* hyphal network. The response was not observed in two different ABA mutants, *aba5-1* and *aba2-1* (Tables 1C,D). These data indicate that part of the threat information systemically traveling from the *A. brassicae* infection site in the leaf to the roots is converted to an ABA stress response in non-infected seedlings after transfer via a *P. indica* hyphal network.

## Discussion

We used *NRT2.5* and *RRTF1* mRNA levels as readouts to monitor radial and axial signal propagation in Arabidopsis leaves and roots following local infections with *A. brassicae* spores. These two genes were chosen because we found that their mRNA levels responded systemically to various stresses in pilot experiments, but they are not directly related to phytohormone responses induced by the pathogen. We are aware that many other genes show a similar regulation pattern. The threat information measured at the level of *NRT2.5* and *RRTF1* is also transferred to neighboring plants when they are connected to the infected plant via a *P. indica* hyphal network. Overall, this information flow is slow (requiring at least 7 days) in this experimental set-up, presumably because spore germination requires time and the systemic stimulus increases only slowly with the progression of disease development. However, since these responses are highly dependent on the amount of spores and culture conditions, our experimental set-up is difficult to compare with studies in which a specific stimulus is applied for a defined period of time to a local tissue (cf. section Introduction). Nevertheless, consistent with previous studies (Dengler, 2006; Kiep et al., 2015), we observed that signal propagation requires a connection to the vascular system and becomes weaker or disappears when the pathogen infection is performed on tissues not directly located on major veins (data not shown). In many studies, the distances between the tissue to which a specific stimulus was applied and that, where the response was measured, were quite short (cf. Jayaraman et al., 2014; Choi et al., 2016, and ref. therein). Those studies allow the identification of components which rapidly transfer the information, while our study measured responses after longer time periods and thus included also major metabolomic and developmental changes. Stimulation of *RRTF1* is generally faster than that of *NRT2.5* (Lezhneva et al., 2014), but the two responses need not necessarily be connected to each other: e.g., fast traveling ROS generated after *A. brassicae* infection could rapidly induce *RRTF1* expression (Khandelwal et al., 2008; Matsuo et al., 2015). We have previously demonstrated that RRTF1 amplifies ROS responses in answer to various stress stimuli (Matsuo et al., 2015), and therefore, it is conceivable that up-regulation of this gene might be part of an alarm system to prepare distal parts of a plant and even neighboring plants to respond more rapidly to upcoming threats. In contrast, the slower response of *NRT2.5* in distal tissue or neighboring plants could be coupled to metabolic changes in the local and distal tissues associated with N metabolism. This transporter is involved in nitrate relocation, and the gene could only be activated when the disease progression after *A. brassicae* infection generates a local nitrate shortage, although a direct involvement of nitrate transporters in defense responses is also discussed (Hu et al., 2009). Several nitrate sensing mechanisms leading to *NRT2.5* regulation are possible (cf. Ho et al., 2009; Hu et al., 2009; Krapp et al., 2014), and a nitrate sensing function has been shown for NRT1.1 (Wang et al., 2009) and proposed for NRT2.1 (Orsel et al., 2004; Little et al., 2005; Ohkubo et al., 2017). Ohkubo et al. (2017) showed that shoot-to-root mobile polypeptides are involved in systemic regulation of nitrogen acquisition. Overall, we observed that the entire plant responds to a local pathogen infection on a leaf or the root. The late time points allow us to assay traveling information even over long distances since progression of disease development causes severe local symptoms that should result in appropriate distal responses.

Both genes are also upregulated in non-infected neighboring plants. Plants often communicate with neighboring plants of the same species to alleviate stresses within genetic relatives by transmitting volatile compounds aboveground or a variety of organic and inorganic compounds belowground (Baldwin and Schultz, 1983; Agrawal, 2000; Song et al., 2010, 2014). The experimental set-up shown in Figure 3A and the split Petri dish experiment (Figure 4) suggested that volatile compounds did not play a role in activating *RRTF1* and *NRT2.5* expression in uninfected seedlings. However, information transfer via common mycrrohizal networks (CMN) is well known (cf. section Introduction), and *P. indica* as an endophyte with the ability to colonize the roots of all plant species investigated so far is an excellent candidate for the transfer of information to neighboring plants even when they are not belonging to the same species. In our experimental set-up, transfer of threat information to neighboring plants via the *P. indica* hyphal connection is supported by experiments in which this connection is disrupted or the performance of the fungal cells is severely impaired by the application of benomyl. Furthermore, it appears that the information transfer is not mediated by just any fungus, since the two candidates chosen, *A. glauca* and *M. mucedo*, cannot replace *P. indica* (Figure 4). The reason for this is unclear, but one might speculate that the cellular connection between fungal and root cells is closer for *P. indica* than the two other investigated fungi. Whether root colonization is important for interplant communication, can be tested by using *P. indica* strains in combination with various (mutant) plants altered in colonization level (Lahrmann et al., 2013; Rafiqi et al., 2013; Akum et al., 2015; Li et al., 2016). Different from CMN with mycorrhizal fungi, invasion of *P. indica* hyphae into root cells is rare (Figure 3B), and most of the hyphae are surrounding the root or are attached to root cells. Therefore, an information transfer via chemical mediators between the fungal and host cell is likely. However, this requires further investigation.

Our data suggest that the neighboring plants respond to the threat information by stimulating accumulation of ABA, a stress hormone that is not up-regulated in infected plants. The most straightforward interpretation of these data is that *A. brassicae* first induces a specific JA stress response in the infected local leaf. This information then travels to systemic roots and is further transferred to neighboring plants via the *P. indica* hyphal network where specific information is converted to general stress information. This interpretation is consistent with the fact that fungi do not contain a JA-specific defense pathway comparable to that in plants, although fungal enzymes can manipulate plant JA metabolism (cf. Patkar et al., 2015). The shift in the hormone levels and expression profiles from JA-responsive genes in the roots of the infected plant to ABA-responsive genes in the roots of the neighboring plant provides us with important tools to further study how this information is translocated though the fungal hyphae. The observation that the JA-specific response to the pathogen in the infected plant is converted to an ABA response in the non-infected neighboring plant is an interesting observation that can be experimentally approached in many ways. ABA has been shown to participate in systemic signaling in response to abiotic stress (Mittler and Blumwald, 2015), participates in the integration of multiple stresses (Nguyen et al., 2016), and cross-talks with defense- and stress-related hormones including JA (Nakata et al., 2013; Verma et al., 2016). Furthermore, the ABA level is up-regulated in response to multiple stresses (e.g., Lievens et al., 2017) and ABA signaling components integrate a wide range of information relevant for stress responses, adaptation and developmental processes. Therefore, it is a good candidate to respond to signals from the fungal hyphae which carry more general stress information due to its contact to a stress-exposed plant. The microbe is unable to understand a specific JA-dependent stress response of the plant, but appears to transmit the threat information, which is then decoded at the receiving plant side and activates a general and not stimulus-related stress response which includes ABA. Interestingly, Hettenhausen et al. (2017) showed recently that host plants connected by *Cuscuta* bridges transmit systemic herbivory signals to unattacked plants. Here interplant signaling is largely dependent on JA signaling and herbivore attack on one host plant elevates defense metabolites in the other connected non-attacked host, resulting in enhanced resistance over longer distances (>100 cm). Although interplant connection occurs via a plant bridge in this study, comparison of this system to Arabidopsis with a *P. indica* hyphal network might help to understand how a specific JA information is further translocated to neighboring plants.

We observed a strong antagonistic effect of JA, JA-Ile, and *cis*-OPDA levels, which are induced by *A. brassicae*, on SA responding to *P. indica* colonization (Figures 5, 6B, cf. also Michal Johnson et al., 2014). In the absence of *P. indica, A. brassicae* induces local and systemic JA, JA-Ile, and *cis*-OPDA accumulation, and this stimulation is also detectable in neighboring plants irrespective of whether they are connected to the infected plants or not. In all cases, the regulation on the phytorhomone level correlated with the response of the respective phytohormone-responsive genes (Figures 6, 7). Suppression of the JA-responsive pathway by SA is predominantly regulated at the level of gene transcription (Van der Does et al., 2010), and not by JA biosynthesis itself, as the SA-mediated suppression of MeJA-induced *PDF1.2* was intact in the JA biosynthesis mutant *aos/dde2* (Leon-Reyes et al., 2010). SA antagonizes JA signaling downstream of COI1, possibly by interfering with JA-regulated transcription factors. Further, several WRKY and TGA transcription factors have been shown to be important for suppression of the JA-responsive pathway by SA (Li et al., 2004, 2006; Pieterse et al., 2012; Gimenez-Ibanez and Solano, 2013; Caarls et al., 2015) have shown that the SA-induced WRKY70 suppressed MeJA-induced *PDF1.2* expression.

Cosme et al. (2016) showed that *P. indica* helps rice plants to tolerate root herbivory through changes in JA signaling. In their study, JA is transported from herbivore-damaged leaves to roots, as first shown by Zhang and Baldwin (1997) using [2-^14^C]JA. Jimenez-Aleman et al. (2015) also demonstrated that a labeled precursor of JA was able to travel from a wounded local tissue to unwounded systemic leaves when applied exogenously. These studies support that jasmonates can be transported and thus transfer information within the plant body. Since jasmonates as well as jasmonate-responsive genes are also up-regulated in non-infested plants not connected to infested plants via a *P. indica* hyphal network, our data suggest that an additional information transfer through the gas phase is likely. However, we failed to identify volatiles responsible for this response. Vahabi et al. (2013) showed that *P. indica* can grow in host roots even when they contain elevated jasmonate levels. This might be important for *P. indica* growth under our conditions and its efficient repression of jasmonate accumulation.

Connecting plants by a *P. indica* hyphal network resulted in elevated ABA levels and expression of ABA-responsive genes in non-infested plants (Figure 6, Table 1). Also Peskan-Berghöfer et al. (2015) showed that elevated ABA levels triggered by osmotic stress promoted *P. indica* colonization of the roots, without impairing plant fitness. Furthermore, sustained exposure to ABA muted defense response in roots and thereby made them more accessible for the mutualist. Again, these data are consistent with our observations that non-infested seedlings with elevated ABA levels contain low jasmonate levels.

Taken together, JA-related threat information can be transferred to as yet unattacked neighboring plants via a *P. indica* hyphal network. We propose that this information transfer is associated with the loss of specific threat information, since the *A. brassicae*-specific JA response induces a more general ABA-dependent stress response in the connected plant. Combination of phytohormone mutants with-omics approaches will help to elucidate how the threat information is transferred from one plant to the other via the *P. indica* hyphal network.

## Author contributions

KV: designed and performed the experiments described in Figure 2A; MR: performed the phytohormone analyses; SS: analysis of hormone-responsive genes; JJ: designed the experiments for systemic signaling; AF: performed the microscopic studies; MM: designed and performed RRTF1 experiments; IS, JG, and RO: supervised the project; RO: wrote the paper. All authors read and approved the final version of the manuscript.

### Conflict of interest statement

The authors declare that the research was conducted in the absence of any commercial or financial relationships that could be construed as a potential conflict of interest. The reviewer PSB and handling Editor declared their shared affiliation.

## Abbreviations

NRT: NITRATE TRANSPORTER
RRTF1: REDOX-RESPONSIVE TRANSCRIPTION FACTOR1
JA: jasmonic acid
ABA: abscisic acid
CMN: common mycorrhizal network
dpi: days past infection.
