# Supplementary material for: Alternaria Brassicae Induces Systemic Jasmonate Responses in Arabidopsis Which Travel to Neighboring Plants via a Piriformsopora Indica Hyphal Network and Activate Abscisic Acid Responses
Source: Front Plant Sci. 2018 May 8;9:626. doi: 10.3389/fpls.2018.00626 (PMC5952412; doi:10.3389/fpls.2018.00626)
Supplement: Supplementary file 1 [file Image_1.PDF]

SUPPLEMENT

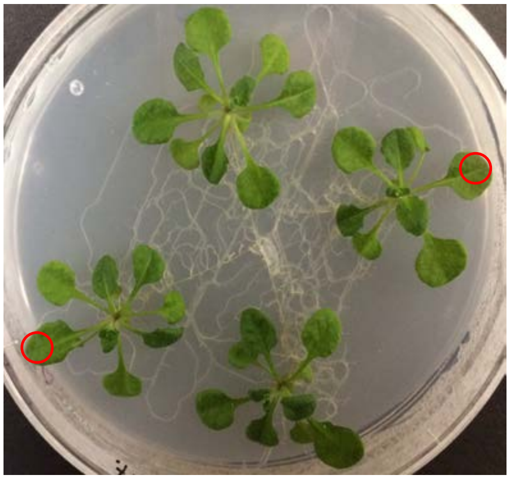

Figure S1. Experimental setup: 4 Arabidopsis seedlings were either grown without *P. indica* (shown here) or on a 1 week-old *P. indica* fungal lawn (not shown because the fungal lawn prevents the detection of the roots on the picture). In each plate, two of the seedlings were inoculated with an *A. brassicae* spore suspension or mock-treated with water (red o), the other two remained untreated.
